# A Novel Method for Intraoral Access to the Superior Head of the Human Lateral Pterygoid Muscle

**DOI:** 10.1155/2014/432635

**Published:** 2014-05-18

**Authors:** Aleli Tôrres Oliveira, Anderson Aparecido Camilo, Paulo Roberto Valle Bahia, Antonio Carlos Pires Carvalho, Marcos Fabio DosSantos, Jorge Vicente Lopes da Silva, André Antonio Monteiro

**Affiliations:** ^1^Orofacial Pain and Masticatory Dysfunction Clinic, Dental Clinic Department, School of Dentistry, Federal University of Rio de Janeiro, Avenida Carlos Chagas Filho 373, Centro de Ciências da Saúde, Bloco K, 2° Andar, Sala 56, Ilha da Cidade Universitária, 21941-902 Rio de Janeiro, RJ, Brazil; ^2^Renato Archer Information Technology Center (CTI), Rodovia Dom Pedro I (SP - 65) Km 143,6, Bairro Amarais, 13069-901 Campinas, SP, Brazil; ^3^Radiology Department, School of Medicine, Federal University of Rio de Janeiro, Avenida Carlos Chagas Filho 373, Ilha da Cidade Universitária, 21941-902 Rio de Janeiro, RJ, Brazil; ^4^Cellular Morphogenesis Laboratory, Biomedical Sciences Institute, Federal University of Rio de Janeiro, Instituto de Ciências Biomédicas, CCS, Bloco F, Ilha da Cidade Universitária, 21949-590 Rio de Janeiro, RJ, Brazil; ^5^Health Sciences Center, Grande Rio University, 1160 Bairro 25 de Agosto, Duque de Caxias, 25071-200 Rio de Janeiro, RJ, Brazil

## Abstract

*Background.* The uncoordinated activity of the superior and inferior parts of the lateral pterygoid muscle (LPM) has been suggested to be one of the causes of temporomandibular joint (TMJ) disc displacement. A therapy for this muscle disorder is the injection of botulinum toxin (BTX), of the LPM. However, there is a potential risk of side effects with the injection guide methods currently available. In addition, they do not permit appropriate differentiation between the two bellies of the muscle. Herein, a novel method is presented to provide intraoral access to the superior head of the human LPM with maximal control and minimal hazards. *Methods.* Computational tomography along with digital imaging software programs and rapid prototyping techniques were used to create a rapid prototyped guide to orient BTX injections in the superior LPM. *Results.* The method proved to be feasible and reliable. Furthermore, when tested in one volunteer it allowed precise access to the upper head of LPM, without producing side effects. *Conclusions.* The prototyped guide presented in this paper is a novel tool that provides intraoral access to the superior head of the LPM. Further studies will be necessary to test the efficacy and validate this method in a larger cohort of subjects.

## 1. Introduction

Functional mandibular movements are the result of precisely coordinated muscle contractions, regulated by a highly refined neurologic system, and with the participation of other craniomandibular structures, including the temporomandibular joints (TMJ), ligaments, and teeth. In this complex arrangement, the mandibular elevator (masseter, temporal, and medial pterygoid) and depressor muscles (lateral pterygoid and supra- and infrahyoid muscles) play an integrated role in mastication and TMJ biomechanics. The lateral pterygoid muscle (LPM) is responsible for three mandibular movements: (1) laterality, caused by unilateral contraction, (2) protrusion, as a result of simultaneous bilateral contraction, and (3) full mouth opening, when its bilateral contraction follows the action of the supra- and infrahyoid muscles. The LPM is anatomically divided in two parts or bellies that coordinate the kinematics of the disc-condyle complex [1]. The superior LPM originates from the infratemporal surface and infratemporal crest of the greater wing of the sphenoid bone and is inserted into the anteromedial aspect of the TMJ capsule and disc; the inferior LPM originates from the lateral surface of the lateral pterygoid plate and is inserted into the pterygoid fovea on the mandibular condyle [2–4]. Due to its complex anatomy and physiology, the human LPM plays an important role in the coordination of the disc-condyle complex motion and mandibular movements. Furthermore, an uncoordinated function of both parts or hyperactivity of its superior belly would contribute to articular disc instability, leading to the very common anterior disc displacement disorders [1, 5–7]. Finally, this muscle has been implicated in other conditions, such as myositis and oromandibular dystonia [6, 8].

Botulinum toxin (BTX) has emerged as a promising therapy to treat myositis as well as oromandibular dystonia. BTX is a potent neurotoxin that acts by producing temporary chemical denervation of skeletal muscles. It has the propriety of inhibiting the release of acetylcholine at the presynaptic junction, producing a transient and dose-dependent reduction of muscle activity, without creating systemic effects [1]. So far, few studies have examined the results of BTX when applied to the human LPM. In the most common technique, the procedure is guided by electromyography (EMG) using an intra- or extraoral access [1, 9–11]. However, it does not permit differentiation between the superior and inferior heads of the LPM. Consequently, the BTX injection may be delivered in both parts of the muscle or only in the inferior LPM. A more invasive approach is the injection guided by arthroscopy [10]. Nonetheless, the reduced space and lack of visibility to the target area makes the differentiation of the superior and inferior parts of the LPM virtually impossible. Moreover, the risk of severe injury or trauma is considered high, due to the close anatomical relation between the LPM and the neurovascular structures located in the infratemporal fossa (ITF) [1, 12]. Therefore, the use of an apparatus for injection guidance to the LPM, with accuracy to target its upper head, would minimize the risks and optimize the results. The advent of modern imaging and rapid prototyping techniques, using computed tomography (CT) [13–16] made possible the development of such device, a customized injection guide to the superior head of the LPM.

## 2. Methods

Virtual modeling was applied, using medical imaging software applications and modeling techniques, such as computer aided design (CAD) that generates STereoLithography (STL) file format. Those techniques were employed to print an injection guide (made of polyamide polymer) in a rapid prototyping machine, to orient the intraoral injection to the superior head of the LPM. In order to test the reliablility of such device, a volunteer took part in the study. All procedures adopted were conducted in accordance with the bioethical rules for studies involving human beings of the World Medical Association (WMA)—Declaration of Helsinki (1990) and the volunteer gave written informed consent prior to the study. The subject recruited had been previously diagnosed with anterior TMJ disc displacement with reduction, on the left side. However, she was not under treatment for her disorder. Her clinical evaluation revealed the presence of reciprocal click and mandibular deviation during the mouth opening. Throughout her clinical exam, she complained of mild pain (3 out of 10 in the visual analogue scale, VAS). She had no history of systemic disorders, recent orofacial surgery, trauma, or CT contraindications.

After the clinical examination, dental impressions were obtained with the irreversible hydrocolloid Hydrogum (Zhermack, Badia Polesine, Italy) and a provisional guide was constructed, using autopolymerizing acrylic resin, JET (Art. Clássico, São Paulo, Brazil), to keep the subject's mandible slightly opened and deflected to the left side. The provisional guide was constructed to provide ample access to the target region (the left upper LPM) during the CT scan. This was also found to be the most appropriate position to access the LPM from an intraoral approach. The provisional appliance was used as a template to the final device modeling and fabrication. In order to obtain satisfactory contrast during the image processing, the provisional guide was filled with a mix of barium sulfate with acrylic resin (1 : 2), following the protocol described in a previous study [17].

**Figure 1:**
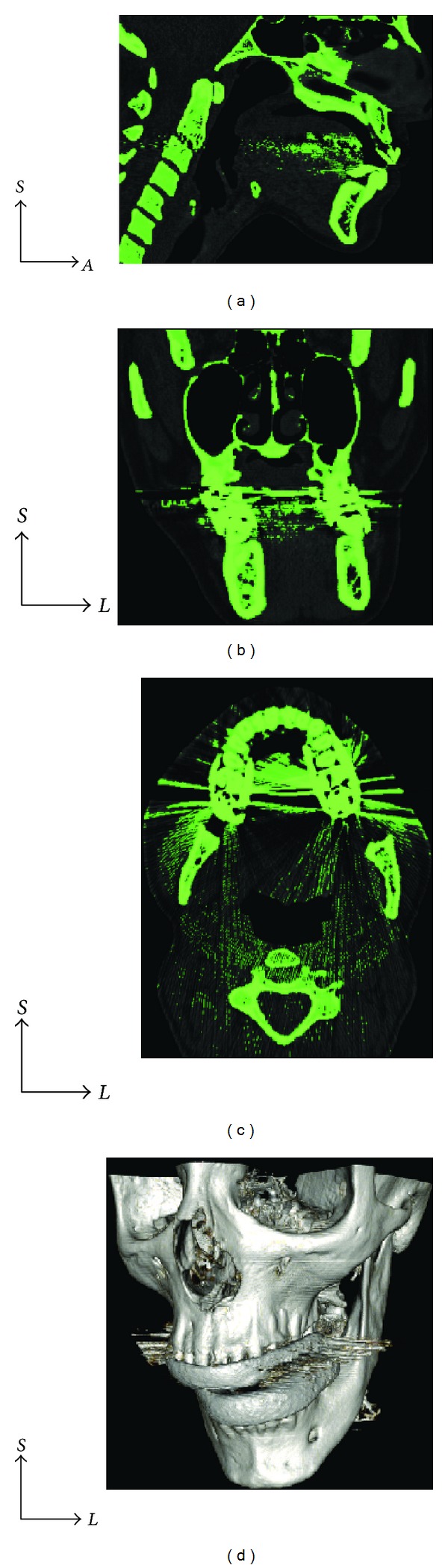
Sagittal (a), coronal (b), and axial (c) CT slices when imported into InVesalius. This software generates 3D reconstructions (d) from 2D DICOM CT or MR images.

With the appliance inserted in the mouth, the volunteer was scanned in a six detectors computed tomograph (Philips Brilliance). The protocol was standardized with the following parameters: Kvp = 120, mA = 250, section thickness = 0,8, reconstruction interval = 0,4, matrix size = 512 × 512, and field of view = 200 mm. All data was saved in a Digital Imaging Communications in Medicine (DICOM) format [18]. The images were imported into InVesalius 3.0 beta3 (CTI, Brazil), an open source software (GNU GPL2) used for reconstruction of computed tomography and magnetic resonance (MR) images [19]. This software generates a 3D reconstruction from a sequence of 2D DICOM files. It uses four windows to view and manipulate the 3D model in the axial, sagittal, and coronal planes. In addition, this program provides filters that are preset for density, thereby creating a semiautomatic segmentation (Figure 1). The Hounsfield scale [12] was further applied to edit, attribute values, and erase imperfections, but maintaining the original anatomy.

**Figure 2:**
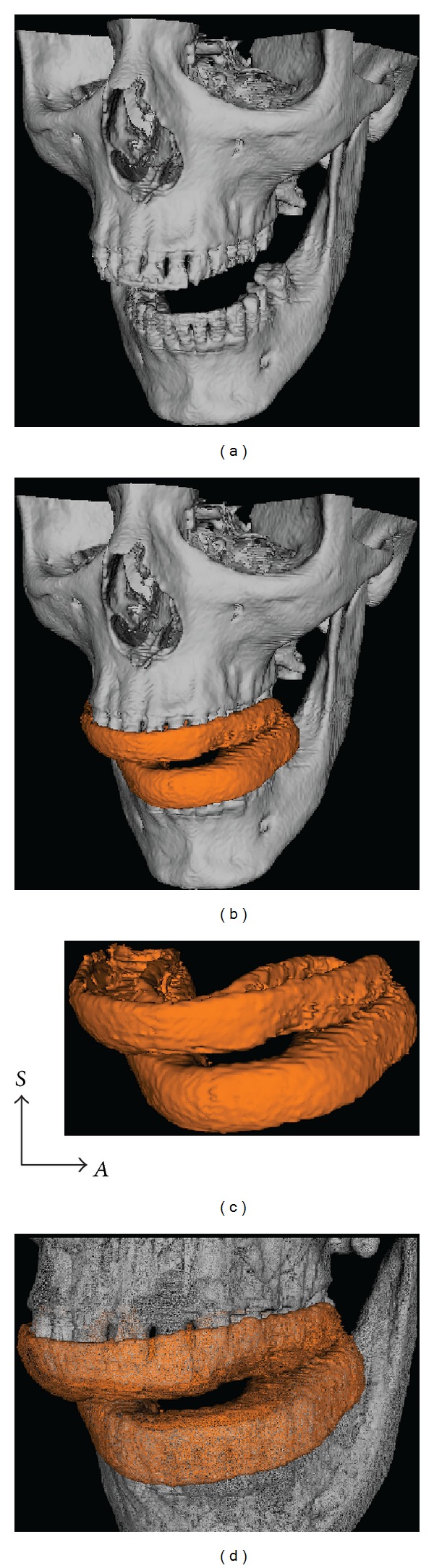
((a)–(d)) The construction of a 3D virtual copy of the provisional guide (orange). (a) 3D reconstruction of the CT images. Notice that the mandible is slighted opened and deflected to the left side. (b) 3D model of the provisional guide (orange) articulated to the dental arches. (c) Provisional guide extracted. (d) Detailed view of the provisional guide adjusted to the upper and lower teeth.

The first challenge was to separate the provisional guide, from the teeth, since artifacts were created during the CT scan, owing to the presence of metallic dental restorations. This semiautomatic separation of soft and hard tissues was achieved with a manual segmentation tool. The result was a 3D provisional guide (Figure 2), which was saved in a STereoLithography (STL) format. STL is a standard file format for rapid prototyping machines composed of a mesh of triangles and normal vectors.

**Figure 3:**
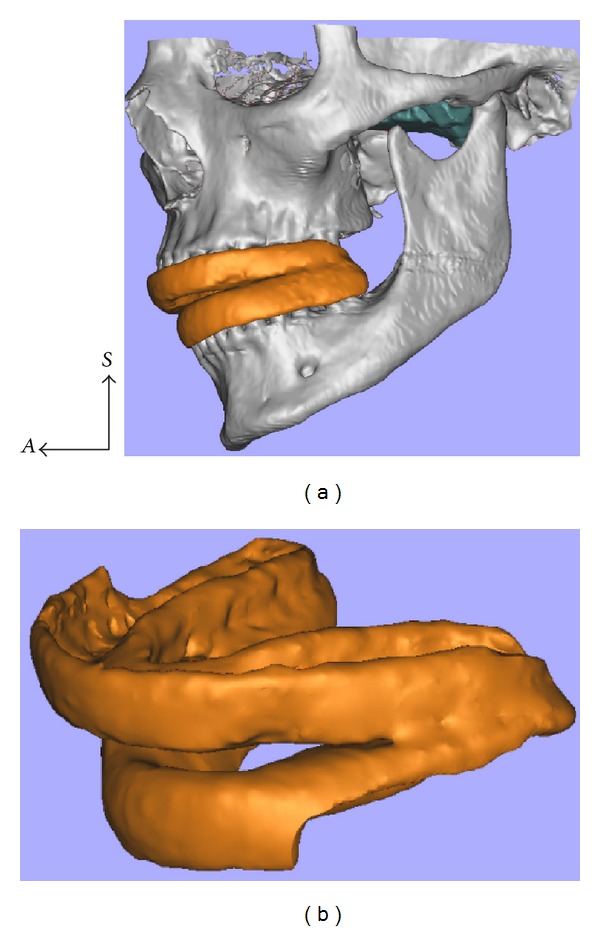
After the segmentation of the superior LPM (green), osseous tissues, and teeth, a 3D virtual model of the permanent guide (orange) was designed and imported into the image processing software Magics STL 17.0. The region close to the junction between the middle part and the posterior two-thirds of the upper LPM was chosen to be the target area (a) and the 3D model of the definitive guide was adjusted to perfectly fit the dental arches of our subject.

**Figure 4:**
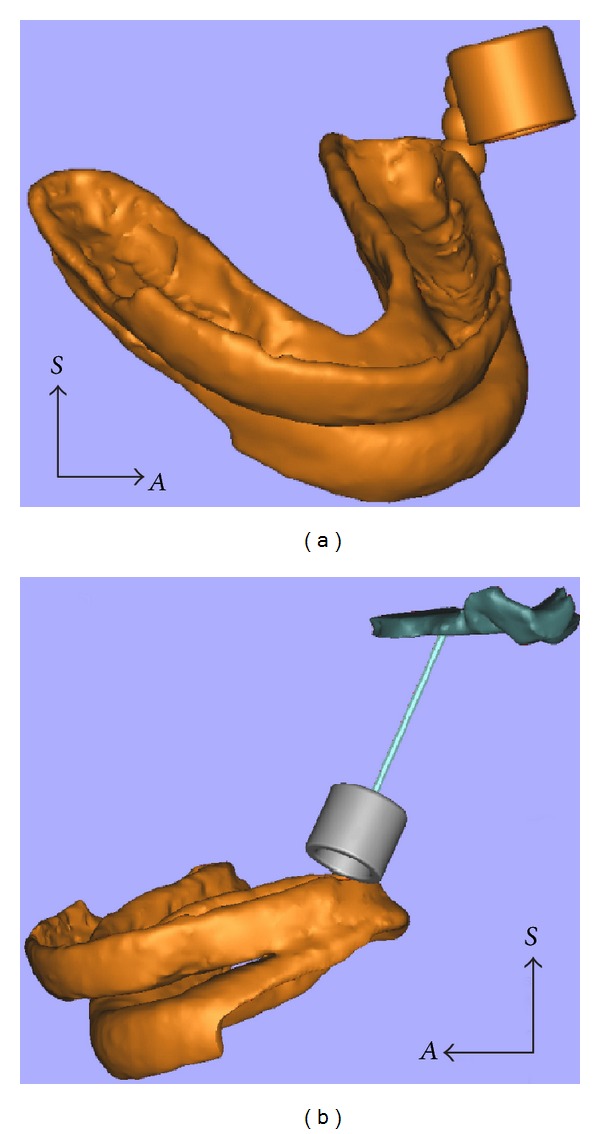
A small tube was virtually built and adapted to the 3D model of the definitive guide to provide access to the superior LPM (a) and a protection was designed in stainless steel, in order to isolate the inner part of this structure (b).

The second challenge was the segmentation of the upper LPM, the most complex phase of the project, since the muscle density is close to the density of the adjoining soft tissue, making their individualization difficult on the image obtained. Therefore, a thorough analysis of the anatomical components of the ITF, specially the upper head of the LPM in the sagittal, coronal, and transverse planes, was critical to build a 3D model of the muscle. Based on a previous report, the average length of the upper LPM is approximately 30 mm [20]. Afterwards, the hard tissue and the 3D virtual model of the definitive guide were designed. The latter was imported into the STL manipulation software Magics STL 17.0 (Materialise, Belgium) (Figures 3(a) and 3(b)) and fine adjustments were made to enable its perfect fit to the dental arches of the volunteer. Those adjustments were necessary due to differences in the long axis angulation of the superior and inferior teeth. Subsequently, a small lumen was designed with the diameter of the needle to guide to the upper LPM. After measuring the size, thickness, and shape of the needle and syringe, a small circular tunnel-shaped structure was constructed (Figure 4(a)).

Considering that the permanent guide would be made of polyamide, a nonmedical grade material, it was prudent to isolate the internal part of the passage that gives access to the LPM, in order to avoid the undesirable transference of this component from the oral mucosa to the ITF by the needle, which could lead to inflammatory reaction in the LPM or other structures in the ITF. This third challenge was solved with the use of the software SolidWorks (Dassault Systèmes SA, France) to design a plug with rounded edges, made of stainless steel, to be inserted into the tube projected to target the superior LPM. All measures were meticulously checked to prevent gaps between the syringe/needle and the small passage just designed, which could interfere in the final position of the needle. Subsequently, the needle trajectory from the intraoral mucosa to the superior portion of the LPM was virtually trained. The infratemporal crest of the sphenoid bone was chosen to be the anatomical region of reference to access the superior LPM, since this is the origin of the upper LPM. After analyzing the CT images, it was decided to direct the needle to the region near the transition between the middle part and posterior two-thirds of the infratemporal crest. This is the area where the upper LPM has its greatest volume. A safety margin was intentionally left, retreating the needle around 2-3 mm from the infratemporal crest.

**Figure 5:**
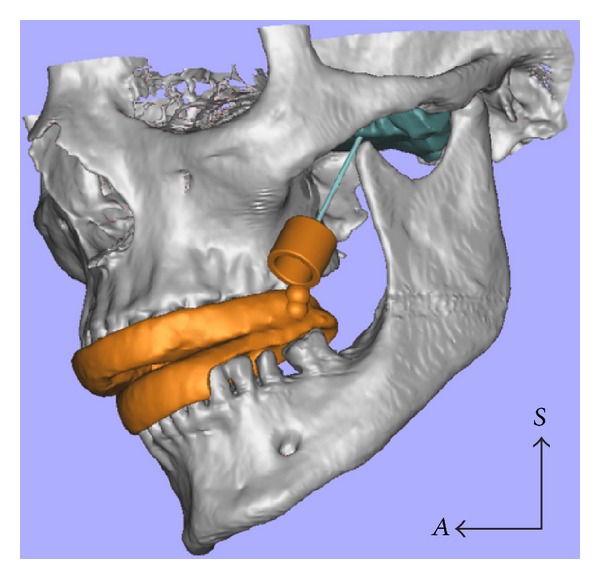
The final project of the upper LPM guide (green) with the virtual needle directed to the upper LPM (green).

**Figure 6:**
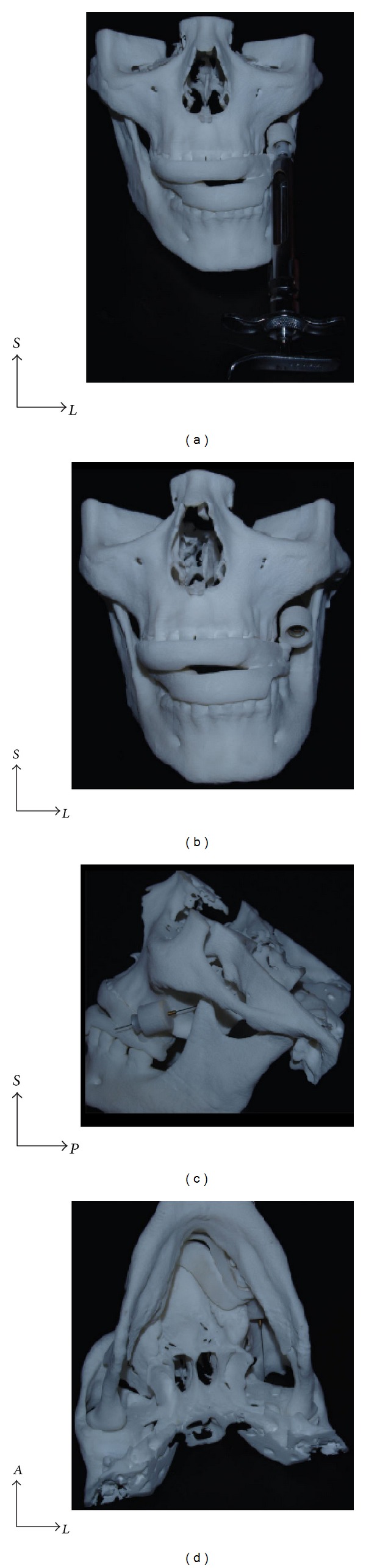
The rapid prototyped guide adjusted to the skull prototype.

Finally, the image of the small circular guide previously projected from the program SolidWorks (Dassault Systèmes SA, France) was imported and aligned with the needle, using the program Magics STL 17.0 (Materialise, Belgium) (Figure 4(b)). In the last phase, all the components of the definitive guide were virtually connected (Figure 5). Upon successful completion of the project, the prototypes of the skull and mandible, as well as the guide for BTX application, were prepared using selective laser sintering (SLS) technology. The unit used to manufacture the device and the biomodel was a Sinterstation 2000 (3D systems, USA). The upper LPM injection guide was then adjusted to the skull prototype and the accuracy of the needle direction was tested. To preliminarily access the clinical reliability of the guide, 2 cc of lidocaine hydrochloride 2%, without vasoconstrictor (Lidostesin DENTSPLY, Brazil), was infiltrated in the left superior head of the LPM of the volunteer with a 27G×1 3/8 (0.4 × 35 mm) dental needle (Terumo, Tokyo, Japan) mounted on a carpule syringe (Figures 6(a)–6(d)).

## 3. Results and Conclusion 

A novel method was developed to produce an injection guide for intraoral access to the superior head of the human LPM, using medical image processing programs and rapid prototyping technology. The guide presented in the current study, resembles the surgical guides commonly used for dental implant placement [21]. Upon the manipulation of the 3D virtual images obtained from a CT scan and the identification of the infratemporal crest of the greater wing of the sphenoid bone, the most consistent anatomical reference to the upper head of the LPM, the layout of the final device was projected. The injection guide was meticulously designed to fit the upper and lower dental arches of our volunteer. The device also contained a narrow passage to orient the needle that was directed to the superior LPM.

The efficacy of BTX application in the LPM to treat anterior TMJ disc displacements has been reported in the literature. However, the LPM access is challenging and there are risks associated with the procedure [1, 4, 6–8]. The goal of such therapy is to abolish spasm of superior head of LPM that prevents the disc from passively retruding with the disc-condyle complex to the TMJ fossa in the mandibular rest position. Instead, the traction provided by the superior head would retain the disc anteriorly, while the condyles would be pushed up and backwards by the powerful mandibular elevator muscles, resulting in uncoordinated disc-condyle kinematics. The thin upper ligament of the bilaminar zone, composed of elastin, would not support the tension provided by the upper LPM spasm, resulting in disc dislocation and clicking sound during its reduction to normal disc-condyle relationship.

Up to now, EMG is the most common technique applied to acces the human lateral pterygoid muscle* in vivo*. Notwithstanding it permits the localization of the LPM; it does not provide the proper differentiation between the superior and inferior parts of the muscle [8]. This characteristic is extremely relevant given that BTX injection in the inferior head of the LPM is invariably associated with temporary limitation of the lateral mandibular movement to the contralateral side [1] and jaw deflection during maximum mouth opening. Although significant adverse events have not been largely described, there is a potential risk when the LPM BTX injection is carried out under EMG guidance, since the anatomical structures are not clearly identified. In fact, even the inferior LPM palpation is difficult and it has not been recommended in the clinical routine examination for temporomandibular disorders (TMDs), since there is a high possibility of false-positive findings [22]. The use of a patient-specific guide to orient the direction of the needle during the BTX injection in the LPM allows the proper differentiation between the superior and inferior LPM. Thus, with this technique it is possible to selectively target the superior LPM, avoiding discomforting or any potential side effect related to the inferior LPM BTX injection. Arthroscopy is another technique commonly applied to guide LPM BTX injection. Nevertheless, it can be considered a more invasive procedure when compared to EMG [10].

Among the structures located in the ITF, the maxillary artery is one of the most important and it should be avoided during LPM injections. A comprehensive knowledge of its variations and its relations to the LPM is highly recommended when planning the procedure. This artery is divided in three portions. Although, in some cases its second part passes deep to the muscle [23], in the most common topographical relationship, it is superficial to the LPM [12], which increases the chance of intravascular injections. Therefore, based on the regional anatomy it is possible to design the access to the superior LPM, preventing traumas to the maxillary artery. The inclusion of an angiotomography exam in our protocol would probably increase the accuracy of our method. However, the risks related to contrast administration and the increase in radiation exposure should be considered.

The guided injection of anesthetics in the upper head of the LPM of the volunteer abolished the signs and symptoms of TMJ clicking, without interfering in the mandibular movements, thus proving to be a reliable and safe method. As expected, the clinical findings obtained were completely reverted in approximately 30 minutes.

Finally, BTX injections have also been applied in the treatment of oromandibular dystonia, which consists of involuntary spasms of masticatory, lingual, perioral, and/or pharyngeal muscles [24]. This condition usually affects both bellies of the LPM and is characterized by mandibular deflection to the side contralateral to the affected muscle. It might be associated with pain, occlusal disorders, difficulties in speech and swallowing, aesthetic alterations, and psychosocial disturbances. [9, 25]. Notwithstanding, the goal of current study was the construction of a methodology to create specific-patient guides to orient the access to the superior head of the LPM; it is also feasible to direct the guide to the inferior LPM, in cases of focal dystonia affecting the lower head of the LPM.

The prototyped guide presented in this study is a reliable tool for accurate and safe intraoral injection in the superior head of the LPM. Further studies will be necessary to test the efficacy and validate the method in a group of subjects with lateral pterygoid muscle disorders affecting the coordination of the TMJ disc-condyle movement.

## Abbreviations

BTX: Botulinum toxin
CAD: Computer aided design
CT: Computed tomography
DICOM: Digital Imaging Communications in Medicine
EMG: Electromyography
GNU GPL2: General Public License, version 2.0
ITF: Infratemporal fossa
LPM: Lateral pterygoid muscle
MR: Magnetic resonance
STL: STereoLithography
TMD: Temporomandibular disorders
TMJ: Temporomandibular joint
VAS: Visual analogue scale
V3: Mandibular nerve (third branch of the fifth cranial nerve)
WMA: World Medical Association
3D: Three-dimensional.
